# Increased LINE-1 expression is associated with neuronal gene network downregulation in the aged mouse prefrontal cortex

**DOI:** 10.3389/fnagi.2026.1961301

**Published:** 2026-10-23

**Authors:** Shinya Fujii, Chu Guo, Yutaka Nakachi, Mohammad Mustakim Billah, Yuko Imamura, Emi Kiyota, Miki Bundo, Kazuya Iwamoto

**Affiliations:** Department of Molecular Brain Science, Graduate School of Medical Sciences, Kumamoto University, Kumamoto, Japan

**Keywords:** DNA methylation, neuroinflammation, retrotransposition, retrotransposons, WGCNA

## Abstract

**Background:**

Aging is associated with epigenetic alterations, neuroinflammation, and progressive decline of neuronal function. Derepression of the retrotransposon LINE-1 (L1) has been implicated in aging and age-related diseases. However, the regional expression of L1 and its relationships with DNA methylation, genomic L1 copy number, and aging-related transcriptional changes in the normal aging brain remain incompletely understood.

**Methods:**

We examined L1 expression in the prefrontal cortex (PFC), hippocampus, and nucleus accumbens of young (17-week-old, *n* = 10) and aged (101-week-old, *n* = 7) male C57BL/6J mice. L1 expression, DNA methylation, and genomic copy number were quantified by RT-qPCR, pyrosequencing, and genomic qPCR, respectively. Transcriptomic alterations in the PFC were analyzed by RNA-seq, followed by weighted gene co-expression network analysis (WGCNA).

**Results:**

L1 expression was significantly increased in the aged PFC across multiple active L1 subfamilies, whereas no significant changes were observed in the other brain regions. Despite elevated L1 expression, no detectable changes were observed in the DNA methylation of active L1 promoters or in genomic L1 copy number between age groups. WGCNA identified three major aging-associated modules. Among these, only the neuronal module showed a significant negative correlation with L1 expression and was enriched for genes involved in synaptic signaling, neuronal development, and neuronal projections.

**Conclusion:**

L1 expression increased in the aged mouse PFC and is associated with coordinated downregulation of neuronal gene networks rather than other major aging-associated transcriptional programs. These findings further characterize the molecular context of increased L1 expression during normal brain aging.

## 1 Introduction

Aging is a progressive biological process accompanied by functional decline across multiple organs and increased susceptibility to disease. At the molecular level, aging is characterized by diverse alterations, including genomic instability, epigenetic dysregulation, and chronic inflammation (López-Otín et al., 2023). Aging is also accompanied by changes in chromatin organization, including heterochromatin relaxation and loss of transcriptional repression within repetitive genomic regions enriched for transposable elements (TEs) (Lee et al., 2020; Wang et al., 2022). Consequently, TEs become transcriptionally activated in aged and senescent cells and have emerged as potential contributors to aging-associated dysfunction (De Cecco et al., 2013; Gorbunova et al., 2021).

The long interspersed nuclear element-1 (LINE-1, L1) is the only autonomously active retrotransposon family in mammalian genomes and accounts for approximately 20% of the mouse and human genomes (Lander et al., 2001; Mouse Genome Sequencing Consortium et al., 2002). Full-length L1 elements contain an internal promoter within the 5′ untranslated region (UTR) and two open reading frames encoding ORF1 and ORF2. These proteins mediate retrotransposition through an RNA intermediate, resulting in the insertion of new L1 copies into the genome (Wallace et al., 2008; Saleh et al., 2019). Although L1 activity is normally suppressed in somatic tissues by epigenetic and cellular defense mechanisms, accumulating evidence suggests that L1 repression becomes compromised during aging and cellular senescence (Gorbunova et al., 2021; Nam et al., 2023).

Recent studies have suggested several mechanisms through which L1 may contribute to aging. Age-associated reductions in the repression of L1 have been linked to alterations in regulators as well as decreased DNA methylation (Van Meter et al., 2014; De Cecco et al., 2019; Simon et al., 2019). In senescent cells, L1 derepression results in the accumulation of L1-derived nucleic acids in the cytoplasm, which activate innate immune pathways and promote the senescence-associated secretory phenotype (SASP) characterized by type I interferon signaling (De Cecco et al., 2019). In addition, L1 RNA has been implicated in heterochromatin erosion and cellular dysfunction in progeroid syndromes (Della Valle et al., 2022). Together, these findings suggest that L1 dysregulation may contribute to aging through epigenetic, inflammatory, and chromatin-related mechanisms. However, whether similar mechanisms accompany L1 activation during normal brain aging remains unclear.

Emerging evidence indicates age-related changes in transposable element regulation in the brain, including increased ORF1 expression and cell-type dependent epigenetic trajectory during aging brain (Bonnifet et al., 2025; Vallés-Saiz et al., 2025; Zeng et al., 2026). These findings raise the possibility that L1 dysregulation may be associated with specific molecular programs in the aging brain. However, it remains unclear whether increased L1 expression is linked to specific aging-related transcriptional programs or instead reflects broader age-associated molecular changes.

In the present study, we investigated age-associated changes in L1 expression in the prefrontal cortex (PFC), hippocampus (Hp), and nucleus accumbens (NAc) of young and aged male mice. We further examined L1 DNA methylation, genomic copy number, and transcriptomic alterations using RNA-seq and weighted gene co-expression network analysis (WGCNA) (Oldham et al., 2008) to clarify the molecular correlates of L1 expression during normal brain aging. Based on previous evidence linking L1 derepression to aging and age-related neuronal alterations, we hypothesized that L1 expression would increase in a region-specific manner during normal brain aging and would be associated with coordinated transcriptional changes related to neuronal function. We further examined whether age-related changes in L1 expression were accompanied by alterations in L1 DNA methylation or genomic copy number.

## 2 Materials and methods

### 2.1 Animals

All animal experiments were conducted in accordance with the National Institutes of Health Guide for the Care and Use of Laboratory Animals. All protocols were approved by the Institutional Animal Care and Use Committee at Kumamoto University (A2023-044), and every effort was made to minimize the number of animals used and their suffering. Male C57BL/6J mice were obtained from a supplier (Charles River Laboratories Japan, Tokyo, Japan). Upon arrival, mice in the aged and control (young) groups were 98-week-old and 14-week-old, respectively, with ten animals in each group. After arrival, the mice were housed individually for approximately three weeks prior to sampling in a temperature- and humidity-controlled and specific pathogen-free environment with a 12 h light/dark cycle (lights on at 7:00) at the Center for Animal Resources and Development of Kumamoto University. Food and water were provided *ad libitum*.

### 2.2 Tissue sampling and extraction of DNA and RNA

An initial sample size of 10 animals per group was selected to balance the need for sufficient biological samples for the planned molecular analyses with the principle of minimizing animal use. No a priori power analysis was performed. Three aged mice died before tissue sampling; therefore, the final sample sizes were 7 aged mice and 10 young adult mice. At the time of sampling, the animals were 101-week-old in the aged group and 17-week-old in the control group (The Jackson Laboratory, 2017; National Institute on Aging, 2026). Under deep anesthesia with isoflurane, mice were decapitated, and their brains were extracted. Brains were placed in a Brain Matrix (catalog no. 68713) (Neuroscience, Inc., Tokyo, Japan), and 1 mm thick coronal sections were prepared using a blade (TCB-100) (Muromachi Kikai, Tokyo, Japan). From these coronal sections, the PFC and NAc regions were bilaterally collected with 1.5 mm diameter biopsy punch with plungers (BPP-15F) (Kai Industries Co., Ltd., Gifu, Japan). The brain was then removed from the matrix, and the right Hp was isolated using spatulas. In addition to brain tissues, tail and liver tissues were also collected. The collected tissues were immediately frozen on dry ice and stored at −80 °C until nucleic acids extraction. DNA and RNA were extracted using the AllPrep Mini Kit (Qiagen, Hilden, Germany) for PFC and Hp samples or the AllPrep Micro Kit (Qiagen) for NAc samples. For RNA samples, on-column DNA digestion was performed using the RNase-Free DNase Set (Qiagen). DNA and RNA concentrations were measured with a Quantus Fluorometer (Promega, Madison, WI, United States). DNA and RNA were extracted from the same PFC tissue samples using the AllPrep DNA/RNA Mini Kit. The resulting DNA and RNA samples were subsequently used for the DNA methylation, genomic L1 copy-number, and RNA expression analyses, respectively.

### 2.3 Reverse transcription-quantitative PCR (RT-qPCR)

A total of 200, 500, and 25 ng of total RNA were used for cDNA synthesis from the PFC, Hp, and NAc, respectively. cDNA was synthesized by reverse transcription (RT) using SuperScript IV Reverse Transcriptase (Thermo Fisher Scientific, Waltham, MA, United States) with Oligo (dT)20 primer. Quantitative PCR (qPCR) was performed in triplicate using Power SYBR Green PCR Master Mix (Thermo Fisher Scientific) with a Quantstudio 5 Real-Time PCR System (Thermo Fisher Scientific). L1 subfamily expression was measured using a subfamily-specific qPCR method adapted from a previously established genomic DNA assay (Kuroki et al., 2020). Gapdh was used as the internal control, and relative expression levels were calculated using the 2^−ΔCt^ method. The primer sequences are listed in the Supplementary Table 1.

### 2.4 Pyrosequencing assay

The DNA methylation level of active mouse L1 subfamilies (TfI, GfII, and A) were measured using a pyrosequencing assay, as described previously (Murata et al., 2017). Briefly, genomic DNA samples were bisulfite-converted and subjected to PCR using a biotinylated primer. After purification with streptavidin-sepharose beads (GE HealthCare, Chicago, IL, United States) and denaturation, the beads were suspended in an annealing buffer containing the sequencing primer. Pyrosequencing was then conducted using a Pyromark Gold Q96 Reagents kit (Qiagen) on a PSQ 96MA instrument (Qiagen). The assay interrogated six CpG sites across the three active L1 subfamilies: two sites in TfI, one site in A, and three sites in GfII. The primers, analyzed sequences, and dispensation orders have been described previously (Murata et al., 2017).

### 2.5 Quantification of L1 DNA copy number by qPCR

DNA copy number of active mouse L1 subfamilies was measured by qPCR as previously reported, with slight modifications (Kuroki et al., 2020). Each reaction used 500 pg of genomic DNA as a template and was performed under the same qPCR conditions as described above. The L1 copy number was normalized using Ct values from the internal control, 5S rRNA. The primer sequences are listed in Supplementary Table 1.

### 2.6 RNA-seq

Total RNA (>1.2 μg per sample) extracted from the PFC was analyzed (Novogene, Tokyo, Japan). RNA quality was assessed using agarose gel electrophoresis, a NanoDrop spectrophotometer (Thermo Fisher Scientific), and an Agilent 2100 Bioanalyzer (Agilent Technologies, Santa Clara, CA, United States). All samples met the quality criteria (OD260/280, 1.8–2.2; OD260/230, ≥2.0; RIN, ≥8.0; 28S:18S, ≥1.0) and were used for strand-specific mRNA library preparation, followed by paired-end 150-bp sequencing on an Illumina NovaSeq 6000 (Illumina, San Diego, CA, United States). Sequencing yielded approximately 20 million read pairs per sample. The raw read data in FASTQ format were firstly assessed with FastQC v0.11.8 and fastq-stats, and then cleaned using Cutadapt v1.18 (Martin, 2011) and Trimmomatic v0.38 (Bolger et al., 2014). The cleaned reads were aligned to the *Mus musculus* GRCm38 reference genome with Ensembl release 102 gene annotation (Howe et al., 2021) using STAR v2.6.0c (Dobin et al., 2013). Gene-level quantification was performed with RSEM v1.3.3 (Li and Dewey, 2011).

### 2.7 RNA-seq data analysis

All analyses were performed using R v4.5.2. Differential expression between aged and control mice was analyzed using edgeR v4.8.2 (Chen et al., 2025). Following the trimmed mean of M-values normalization (Robinson and Oshlack, 2010), quasi-likelihood generalized linear models were fitted and tested using quasi-likelihood F-tests. Differentially expressed genes (DEGs) were defined as genes with an FDR < 0.05 and a between-group mean difference in log_2_(transcripts per million [TPM] + 1) deviating by more than three standard deviations from the genome-wide mean difference.

Total L1 expression levels were quantified using TEtranscripts (Jin et al., 2015). Counts per million (CPM) values for all L1 features were summed for each sample and compared between groups using an unpaired two-sided Student’s *t*-test. Additionally, the expression of full-length and intact L1s (retrotransposition-capable L1s, rc-L1s) was quantified using MORE-RNAseq (Nakachi et al., 2025), which utilizes a GTF file containing manually curated rc-L1s retrieved from L1Base2 (Penzkofer et al., 2017).

Co-expression modules were identified from the TPM matrix using WGCNA v1.74 (Langfelder and Horvath, 2008). A soft-thresholding power of 14 was applied, and modules were defined from topological overlap matrix (TOM)-based hierarchical clustering using dynamic tree cutting (deepSplit = 2; minimum module size = 20) (Langfelder and Horvath, 2008). For each module, the module eigengene, defined as the first principal component summarizing the expression profiles of genes within that module, was calculated and correlated with experimental traits using Pearson correlation. Gene Ontology (GO) and Kyoto Encyclopedia of Genes and Genomes (KEGG) enrichment analyses were performed using clusterProfiler v4.18.4 (Wu et al., 2021), with Ensembl IDs converted to Entrez IDs using org.Mm.eg.db v3.22.0. Redundant GO terms were removed using a semantic-similarity cutoff of 0.7 (Yu et al., 2010). DEG enrichment within modules was assessed using Fisher’s exact test.

## 3 Results

### 3.1 L1 expression increases in the PFC of aged mice

L1 mRNA expression was quantified by RT-qPCR using three sets of primers targeting consensus sequences within the 5’UTR, ORF1, and ORF2 regions (Figure 1A). In the PFC, aged mice (101-week-old, *N* = 7) showed significantly higher L1 expression than young mice (17-week-old, *N* = 10) across all three regions examined (Figure 1B). In contrast, age-associated increases were minimal in the Hp and NAc. To determine whether this increase was specific to particular L1 subfamilies, we next measured the expression levels of individual L1 subfamilies (Figure 2A). In the PFC, expression of all examined subfamilies, including A, Gf, and Tf, was significantly increased in aged mice (Figure 2B). No significant changes were detected in the Hp or NAc (Figures 2C,D).

**Figure 1:**
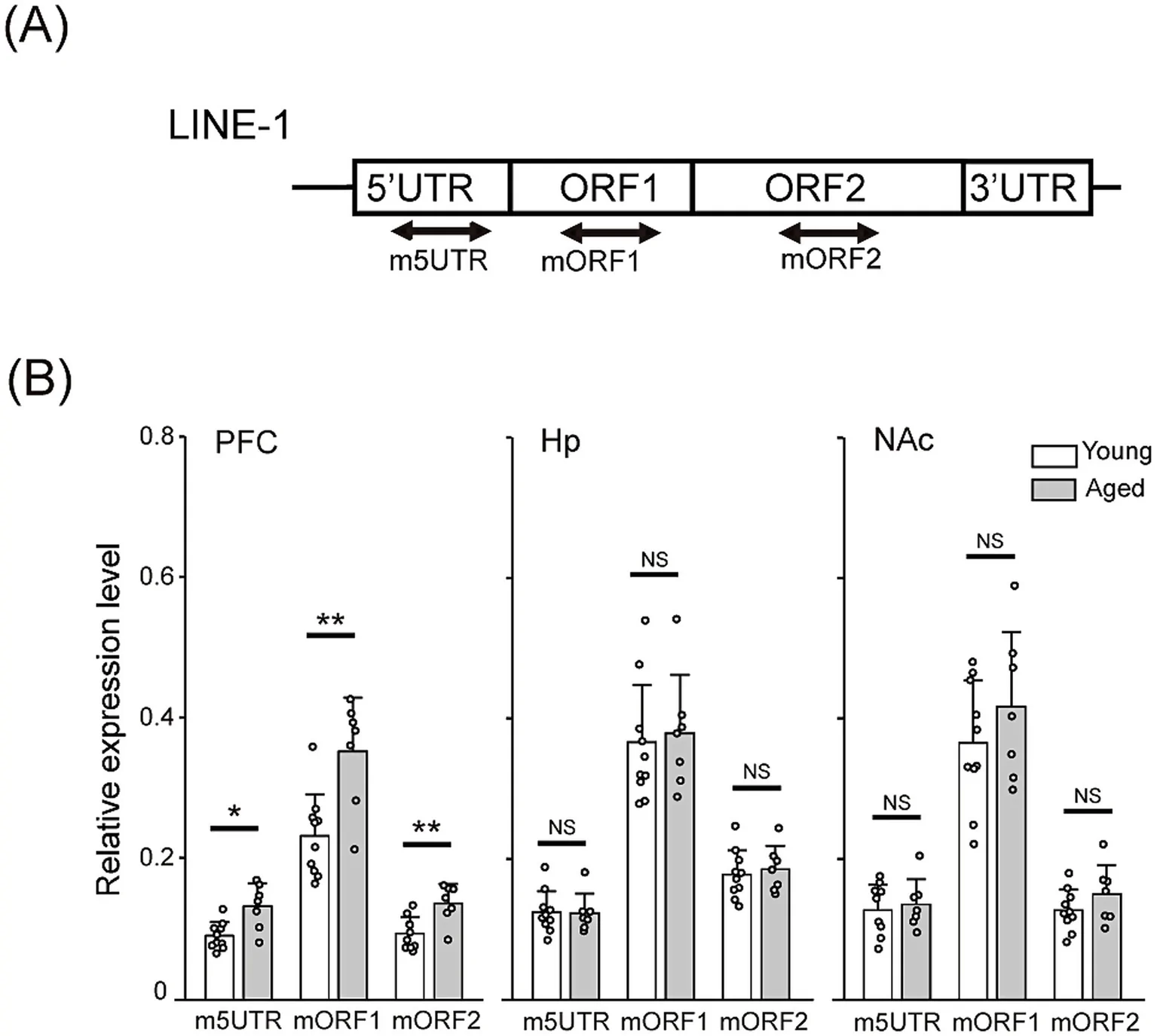
Increased L1 expression in the prefrontal cortex of aged mice. **(A)** Locations of PCR amplicons used for analysis of conserved mouse L1 sequences. **(B)** Relative expression levels of L1 measured by RT-qPCR in the prefrontal cortex (PFC), hippocampus (Hp), and nucleus accumbens (NAc) of young (*N* = 10) and aged (*N* = 7) mice. Expression levels were measured by RT-qPCR using Gapdh as an internal control. Bars represent mean ± SD, and individual data points are shown (young, white bars; aged, gray bars). Statistical significance was evaluated using Welch’s *t*-test. *, *P* < 0.05; **, *P* < 0.01; NS, not significant.

**Figure 2:**
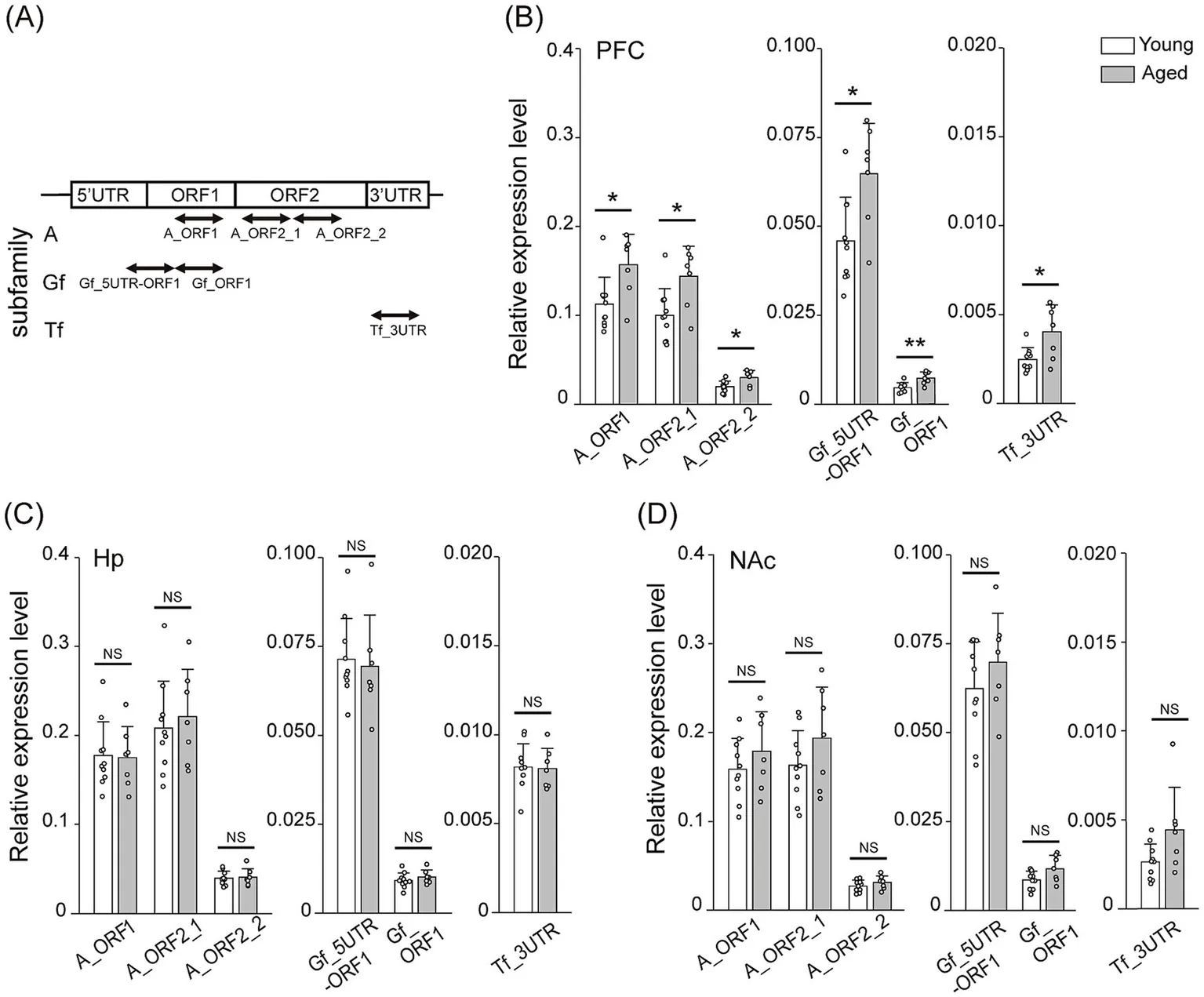
Expression of major L1 subfamilies in the brains of young and aged mice. **(A)** Locations of PCR amplicons used for L1 subfamily-specific analysis. **(B–D)** Relative expression levels of L1 subfamilies in the prefrontal cortex (PFC) **(B)**, hippocampus (Hp) **(C)**, and nucleus accumbens (NAc) **(D)** of young (*N* = 10) and aged (*N* = 7) mice. Expression levels were quantified by RT-qPCR using Gapdh as an internal control. Bars represent mean ± SD, and individual data points are shown. Statistical significance was assessed using Welch’s *t*-test. *, *P* < 0.05; **, *P* < 0.01; NS, not significant.

### 3.2 Expression of genes involved in aging and L1 regulation in aged mouse brain

We measured the expression levels of genes related to aging and the regulation of L1 activity by RT-qPCR (Figure 3). Bdnf and its receptor TrkB are well known for their roles in brain aging and AD (Tapia-Arancibia et al., 2008; Nagahara et al., 2009; Lu et al., 2013; Fonseca-Gomes et al., 2024). Consistent with previous reports of age-associated reductions, we observed significantly decreased Bdnf expression in the PFC and NAc (Figure 3A). Next, we measured the expression levels of Trex1, Rb1, Yy1, and Bmi1, which have been implicated in L1 regulation and/or aging-related chromatin regulation. The expression levels of Trex1 and Bmi1 were significantly higher in aged mice across all three brain regions. Rb1 and Yy1 showed significantly higher expression in the NAc of aged mice (Figure 3B). Given the established roles of the Sirtuins in genome maintenance, chromatin regulation, and aging, together with evidence that Sirt6 directly represses L1 transcription (Haigis and Sinclair, 2010; Van Meter et al., 2014; Gorbunova et al., 2021), we also examined the expression of Sirtuin family genes. In the PFC and Hp, Sirt3 was significantly increased in aged mice (Figure 3C). In the NAc, Sirt1, Sirt4, and Sirt5 were significantly increased. Overall, aging was associated with increased expression of several L1 regulatory genes and sirtuin family members, with expression patterns varying across brain regions.

**Figure 3:**
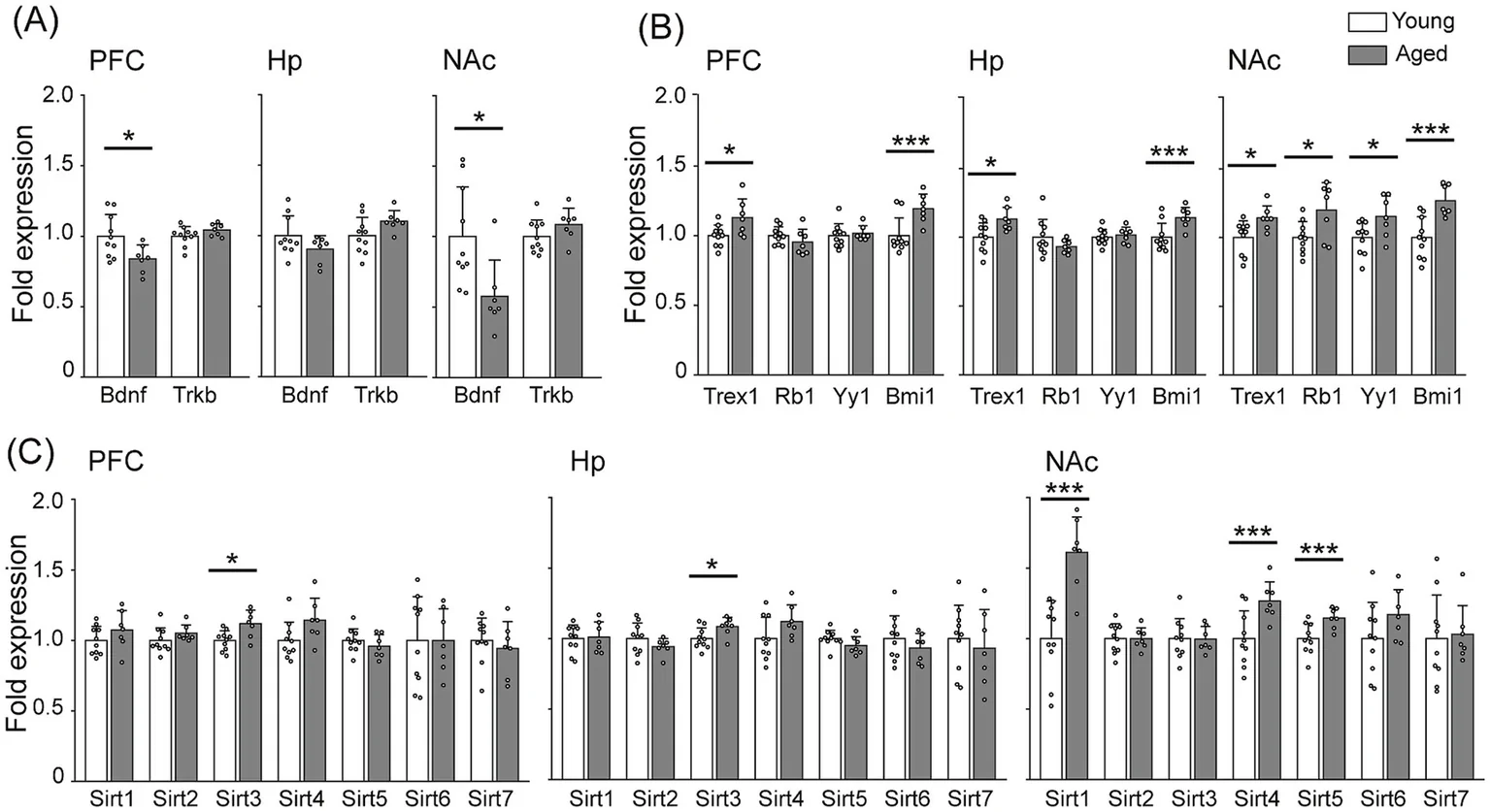
Expression of aging- and L1 regulation-related genes in the brains of young and aged mice. **(A)** Relative mRNA expression levels of Bdnf and Trkb. **(B)** Relative mRNA expression levels of Trex1, Rb1, Yy1, and Bmi1, genes implicated in the regulation of LINE-1 activity. **(C)** Relative mRNA expression levels of Sirt1 to Sirt7. Gene expression levels were quantified by RT-qPCR using Gapdh as an internal control and are presented relative to the mean expression level of the young group in each brain region, which was set to 1. Data are presented as mean ± SD with individual animals shown as dots (young, white bars; aged, gray bars). Statistical significance was assessed using Welch’s *t*-test. *, *P* < 0.05; ***, *P* < 0.005.

### 3.3 DNA methylation levels of L1 are not altered in the PFC

To investigate whether the increased L1 expression observed in the PFC of aged mice was accompanied by changes in DNA methylation at selected CpG sites within the 5′-UTR of active L1 subfamilies, we measured DNA methylation levels of three active L1 subfamilies using bisulfite PCR followed by pyrosequencing (Murata et al., 2017) (Figure 4A). No significant changes in DNA methylation levels were detected at the CpG sites examined in the aged mice (Figure 4B).

**Figure 4:**
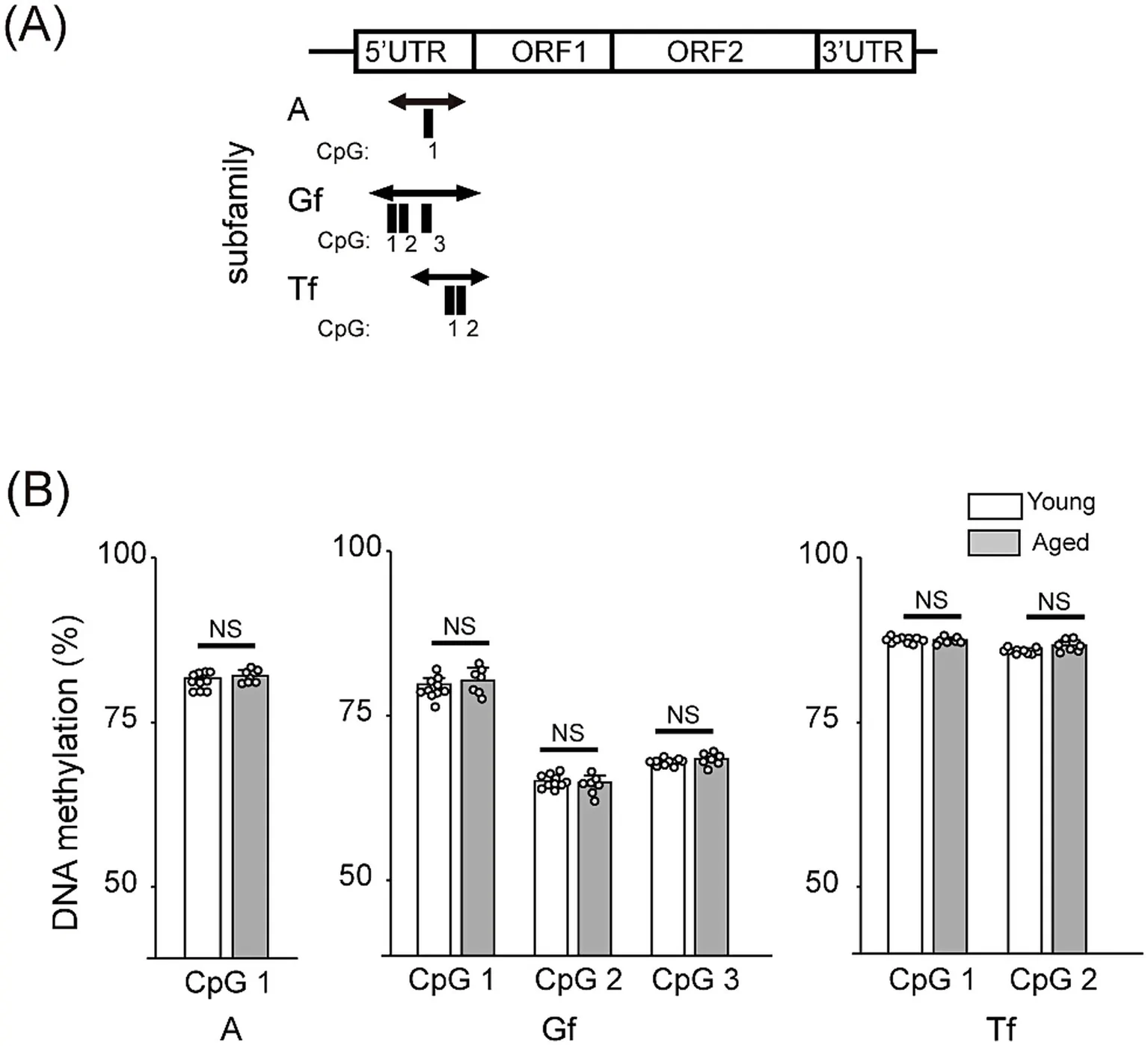
DNA methylation levels of active L1 subfamilies in the PFC of young and aged mice. **(A)** Schematic representation of the bisulfite PCR amplicons and CpG sites analyzed in the 5’UTR regions of the active L1 subfamilies. **(B)** DNA methylation levels at individual CpG sites of the L1 subfamilies. DNA methylation levels were determined by bisulfite PCR followed by pyrosequencing. Data are presented as mean ± SD with individual animals shown as dots (young, white bars; aged, gray bars). Statistical significance was determined using Welch’s *t*-test. NS, not significant.

### 3.4 L1 DNA copy number is not altered in the prefrontal cortex

We next evaluated L1 copy number in the PFC. Genomic DNA extracted from the same cohort of young and aged mice was analyzed by qPCR as previously described (Kuroki et al., 2020). We observed no significant differences in L1 copy number between young and aged mice, either for conserved L1 sequences or for the active L1 subfamilies (Figures 1A, 2A, 5). Rather, a slight decrease was observed when L1 copy number was normalized to genomic DNA from tail or heart (Supplementary Figure 1).

**Figure 5:**
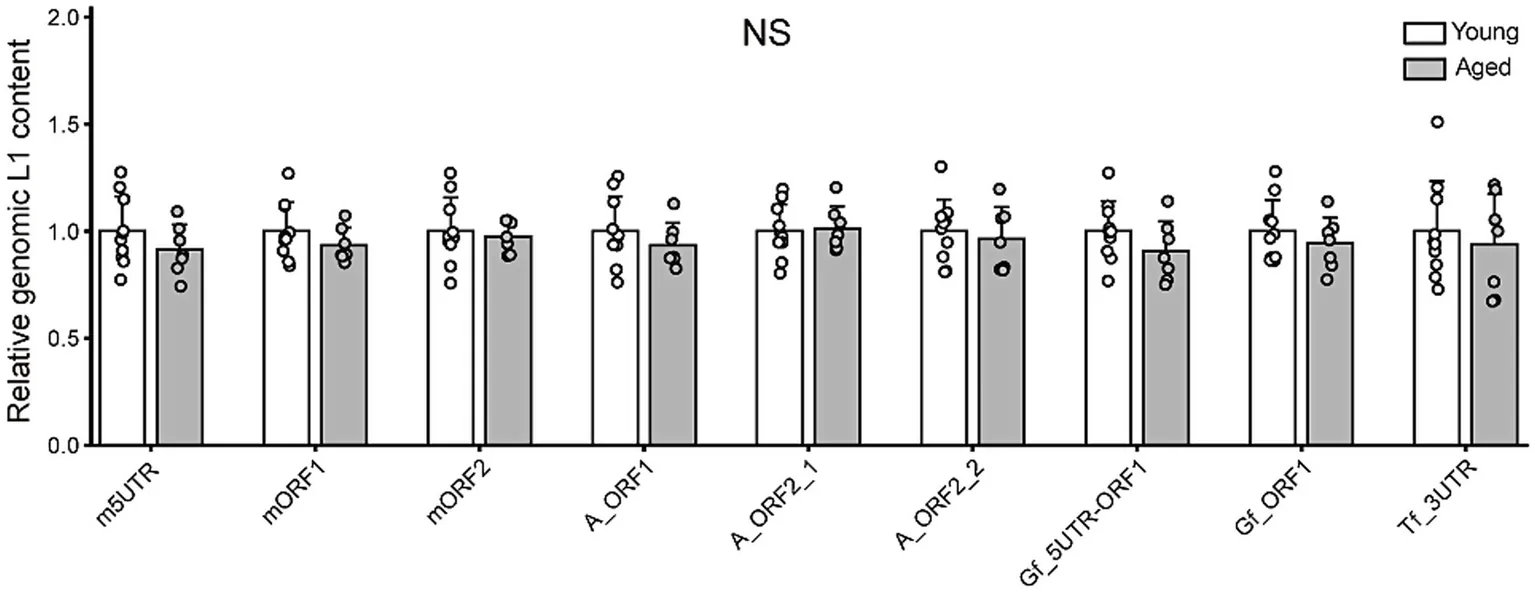
Relative L1 genomic copy number in the PFC. Genomic L1 copy number was quantified by qPCR using primer sets targeting the conserved L1 regions (Figure 1A) and active L1 subfamilies (Figure 2A). L1 copy number was normalized to 5S rRNA. Values are expressed relative to the mean of the young group, which was set to 1. Data are presented as mean ± SD with individual animals shown as dots (young, white bars; aged, gray bars). Statistical significance was determined using Welch’s *t*-test. NS, not significant.

### 3.5 RNA-seq reveals transcriptomic alterations associated with increased L1 expression during aging

To characterize transcriptomic alterations associated with increased L1 expression during aging, we performed RNA-seq using the same PFC samples analyzed in the preceding experiments. Expression changes identified by RT-qPCR were reproduced in the RNA-seq data, including decreased expression of Bdnf and increased expression of Bmi1 and Sirt3 in aged mice (Supplementary Figure 2). Increased L1 expression in aged mice was confirmed by TEtranscripts analysis (Jin et al., 2015), which quantifies transposable element expression (Supplementary Figure 3). We additionally quantified the total expression levels of full-length and intact retrotransposition-capable L1s (rc-L1s), including the Tf, Gf, and A subfamilies, using MORE-RNAseq (Nakachi et al., 2025). Expression levels obtained by the two approaches were positively correlated (*r* = 0.56, *P* = 0.02). We identified a total of 570 DEGs, comprising 422 upregulated and 148 downregulated genes in aged mice.

### 3.6 Identification of aging-associated gene co-expression modules

To investigate coordinated transcriptional programs underlying the observed aging-associated gene expression changes, we performed WGCNA using all expressed genes. This analysis identified 20 co-expression modules (Figure 6A, Supplementary Table 2). Module–trait correlation analysis demonstrated that three modules were significantly associated with aging after FDR correction. The brown module showed a strong negative correlation with aging (*r* = −0.903, FDR-adjusted *P* = 1.39 × 10^–5^), whereas the turquoise (*r* = 0.724, *P* = 0.0102) and lightgreen (*r* = 0.637, *P* = 0.0398) modules were positively correlated with aging. To determine whether the identified modules reflected transcriptional changes occurring during aging, we examined the enrichment of aging-associated DEGs within each module using Fisher's exact test. Significant enrichment was observed exclusively in the above three aging-associated modules (Figure 6B). Aging-associated DEGs were significantly enriched in the brown (139 genes; fold enrichment [FE] = 2.6), turquoise (328 genes; FE = 3.9), and lightgreen (41 genes; FE = 2.4) modules, with all enrichments remaining significant after FDR correction. Together, these modules contained 89.1% of all aging-associated DEGs, capturing the major transcriptional changes associated with brain aging. Functional enrichment analysis demonstrated distinct biological functions for each aging-associated module (Figure 6C, Supplementary Table 2). The brown module was enriched for genes involved in neuronal and synaptic function, including synapse organization, dendrite development, and neurotransmitter signaling. The turquoise module was enriched for ribosomal and stress response including, including ribosome biogenesis, protein quality control, oxidative stress, and antiviral responses. The lightgreen module was characterized by genes associated with myelination and glial activation, including oligodendrocyte differentiation, axon ensheathment, and leukocyte migration.

**Figure 6:**
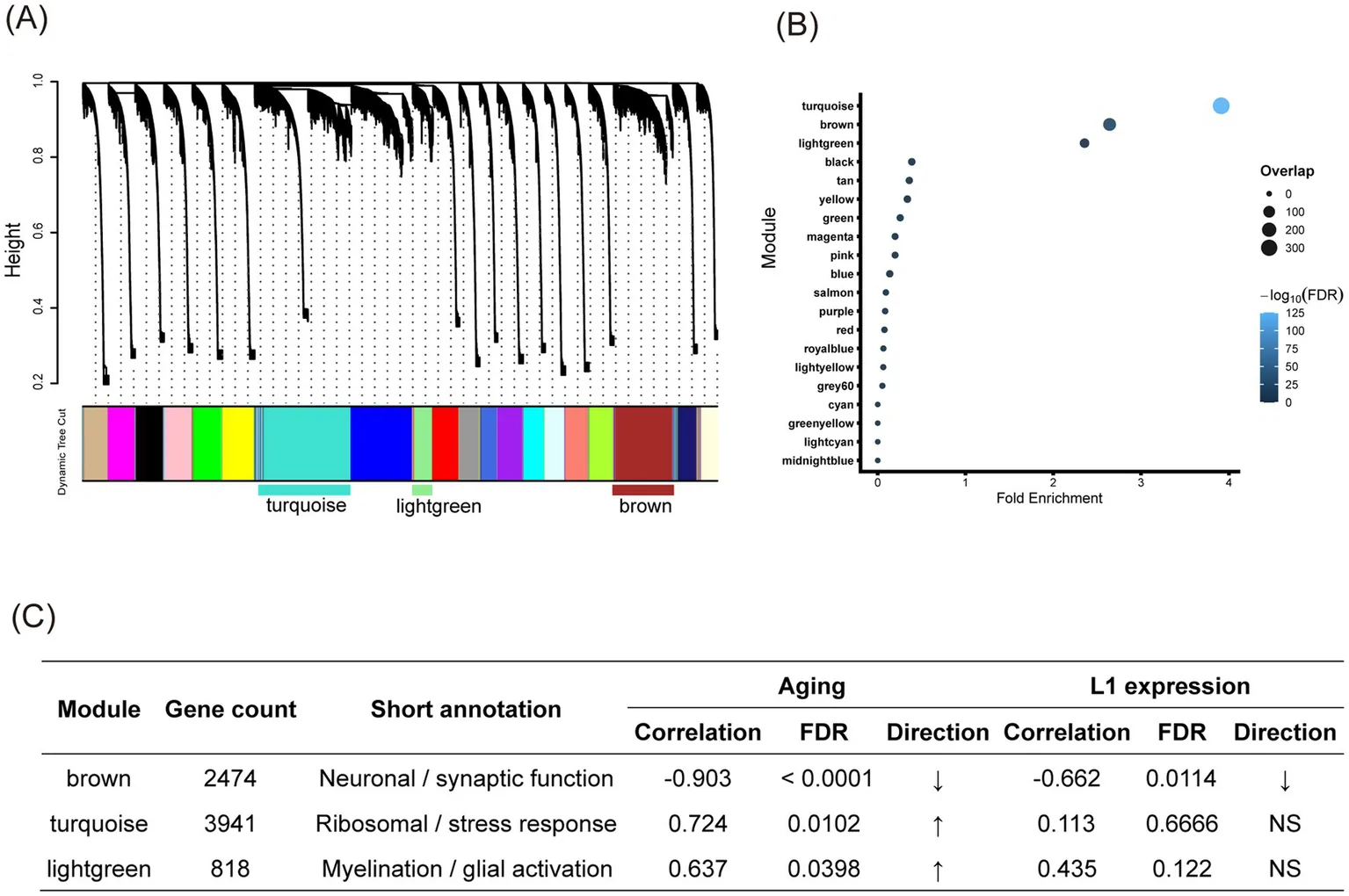
Identification and characterization of aging-associated gene co-expression modules and their associations with L1 expression. **(A)** Hierarchical clustering dendrogram of genes based on topological overlap, with module assignments indicated by the color bar. Detailed annotations of each module are found in Supplementary Table 2. **(B)** Enrichment of aging-associated differentially expressed genes (DEGs) in each WGCNA module, assessed using Fisher’s exact test. Dot size represents the number of overlapping genes, and color indicates −log_10_(FDR). Significant enrichment was observed in the brown, turquoise, and lightgreen modules. **(C)** Summary of the aging-associated modules, including gene counts, functional annotations, and correlations of module eigengenes with aging and L1 ORF2 expression. Aging was coded as young = 0 and aged = 1. Arrows indicate the direction of association, and NS indicates not significant.

### 3.7 L1 expression is preferentially associated with the neuronal co-expression module

We examined the correlations between module eigengenes and L1 expression, which was quantified by RT-qPCR measurement of ORF2 expression. ORF2 was selected because it serves as a useful marker of potentially active L1 elements, and its expression measured by RT–qPCR was strongly correlated with L1 expression estimated using TEtranscripts (*r* = 0.81, *P* = 7.1 × 10^–5^; Supplementary Figure 3). Among the three aging-associated modules, only the brown module showed a significant correlation with ORF2 expression (*r* = −0.662, FDR-adjusted *P* = 0.0114), whereas the turquoise and lightgreen modules showed no significant association. Likewise, no significant correlations were observed between ORF2 expression and any of the other identified modules.

## 4 Discussion

In the present study, we demonstrated that L1 expression is selectively increased in the PFC of aged mice, whereas only minimal changes were observed in the Hp and NAc. By integrating RNA-seq with WGCNA, we identified three major transcriptional programs underlying brain aging: coordinated downregulation of neuronal gene expression, activation of cellular stress-related pathways, and increased glial-associated gene expression. Notably, among these aging-associated modules, only the neuronal co-expression module was significantly associated with L1 expression. Despite elevated L1 expression, neither global hypomethylation of L1 promoter regions nor increased L1 genomic copy number was detected. Together, the inverse association between L1 expression and the neuronal co-expression module may reflect a shared age-related regulatory environment in which neuronal transcriptional programs are reduced while L1 repression is relaxed. However, the present data do not establish whether neuronal transcriptional changes contribute to L1 derepression or result from increased L1 activity.

Aging-associated DEGs were concentrated within three co-expression modules, indicating that brain aging is driven by coordinated transcriptional programs rather than isolated gene expression changes. This observation is consistent with previous network-based analyses demonstrating that aging involves coordinated remodeling of neuronal and non-neuronal transcriptional networks (Ham and Lee, 2020; Kumar et al., 2024). This network-based approach enabled us to evaluate whether increased L1 expression was associated with specific aging-related transcriptional programs. Among the three aging-associated modules, only the neuronal module was significantly associated with L1 expression. These findings suggest that L1 derepression is preferentially linked to coordinated downregulation of neuronal gene networks rather than to broader aging-associated transcriptional changes. Previous studies have shown that L1 derepression activates the cGAS–STING pathway and promotes age-associated inflammation (De Cecco et al., 2019; Simon et al., 2019). In the present study, transcriptomic analysis revealed activation of stress-response pathways and increased expression of immune- and glia-associated genes in aged PFC. However, these transcriptional programs were not significantly associated with L1 expression, indicating that L1 derepression is more closely linked to neuronal transcriptional changes than to glial or inflammatory responses.

L1 expression level quantified using TEtranscripts was well correlated with the expression of retrotransposition-capable L1s (rc-L1s) quantified using MORE-RNAseq, suggesting that rc-L1 expression was associated with the overall increase in L1 expression. However, L1 upregulation in the aged PFC was not accompanied by reduced methylation at active L1 promoters or an increase in genomic L1 copy number. Previous studies have shown that age-associated DNA hypomethylation varies considerably among tissues and repetitive element classes, with L1 methylation remaining relatively stable in several tissues, including the brain (Jintaridth and Mutirangura, 2010; Ryu et al., 2011; Unnikrishnan et al., 2018). This tissue- and context-dependent regulation of L1 methylation is particularly relevant when interpreting our findings, because DNA methylation was assessed using bulk PFC DNA. Consequently, cell-type-specific changes in L1 methylation may have been diluted or masked by the analysis of heterogeneous tissue. Moreover, the pyrosequencing assay examined selected CpG sites within the 5′-UTR of three active L1 subfamilies rather than the entire L1 promoter landscape. Thus, the absence of significant differences at the CpG sites examined does not exclude more localized or cell-type-specific alterations in L1 methylation. Consistent with this possibility, a recent single-cell multi-omic analysis of the aging mouse brain demonstrated that transposon demethylation occurs in a highly cell type-specific manner across neuronal and glial populations (Zeng et al., 2026). Therefore, the absence of detectable changes in bulk PFC does not exclude localized hypomethylation in specific cell populations. Likewise, rare somatic L1 insertions occurring in a limited number of cells would be difficult to detect using bulk copy number assays. Future single-cell approaches will be required to determine whether cell type-specific L1 derepression is accompanied by local DNA demethylation and low-frequency somatic retrotransposition events during brain aging.

Several genes, including Trex1, Rb1, Yy1, and Bmi1, have previously been implicated in L1 regulation and have been associated with reduced expression during aging (Athanikar et al., 2004; Stetson et al., 2008; Abdouh et al., 2012; De Cecco et al., 2019; Saha et al., 2024). However, we did not observe reduced expression of these genes in the aged PFC. Instead, their expression was maintained or upregulated in the aged mouse brain, with expression patterns varying across brain regions. These findings suggest that the transcriptional regulation of genes involved in L1 control differs between normal brain aging and pathological aging or senescent tissues. However, because the present study assessed only mRNA expression, it remains possible that alterations in protein abundance, subcellular localization, or enzymatic activity contribute to L1 derepression during aging.

Several limitations should be acknowledged. First, the sample size was relatively modest, particularly in the aged group (*n* = 7), limiting statistical power for correlation analyses. Second, the present study is based primarily on transcriptomic associations and therefore cannot establish causal relationships between L1 derepression and neuronal dysfunction. Third, because our analyses were performed using bulk PFC tissue, we could not identify the specific cell types responsible for increased L1 expression or the observed aging-associated transcriptional changes. Finally, although no increase in genomic L1 copy number was detected, rare somatic retrotransposition events cannot be excluded. Future studies combining single-cell transcriptomics, chromatin profiling, and experimental manipulation of L1 activity will be important for elucidating the molecular mechanisms linking L1 derepression to neuronal aging.

An additional limitation is that only male mice were included in the present study. Thus, it remains unclear whether the age-related increase in L1 expression observed in the PFC is also present in females. Sex-dependent differences in aging-related molecular regulation could potentially influence L1 expression during brain aging, and future studies including both sexes will be needed to address this possibility.

## 5 Conclusion

L1 expression is increased in the prefrontal cortex during normal brain aging, where it is preferentially associated with the neuronal transcriptional program among the major transcriptional changes occurring during brain aging.

## Data Availability

The datasets presented in this study can be found in online repositories. The names of the repository/repositories and accession number(s) can be found at: https://www.ncbi.nlm.nih.gov/geo/query/acc.cgi?acc=GSE337420 with GEO accession GSE337420.
