# Supplementary material for: Increased LINE-1 expression is associated with neuronal gene network downregulation in the aged mouse prefrontal cortex
Source: Front Aging Neurosci. 2026 Oct 23;18:1961301. doi: 10.3389/fnagi.2026.1961301 (PMC13739680; doi:10.3389/fnagi.2026.1961301)
Supplement: Supplementary file 1 [file Data_Sheet_1.PDF]

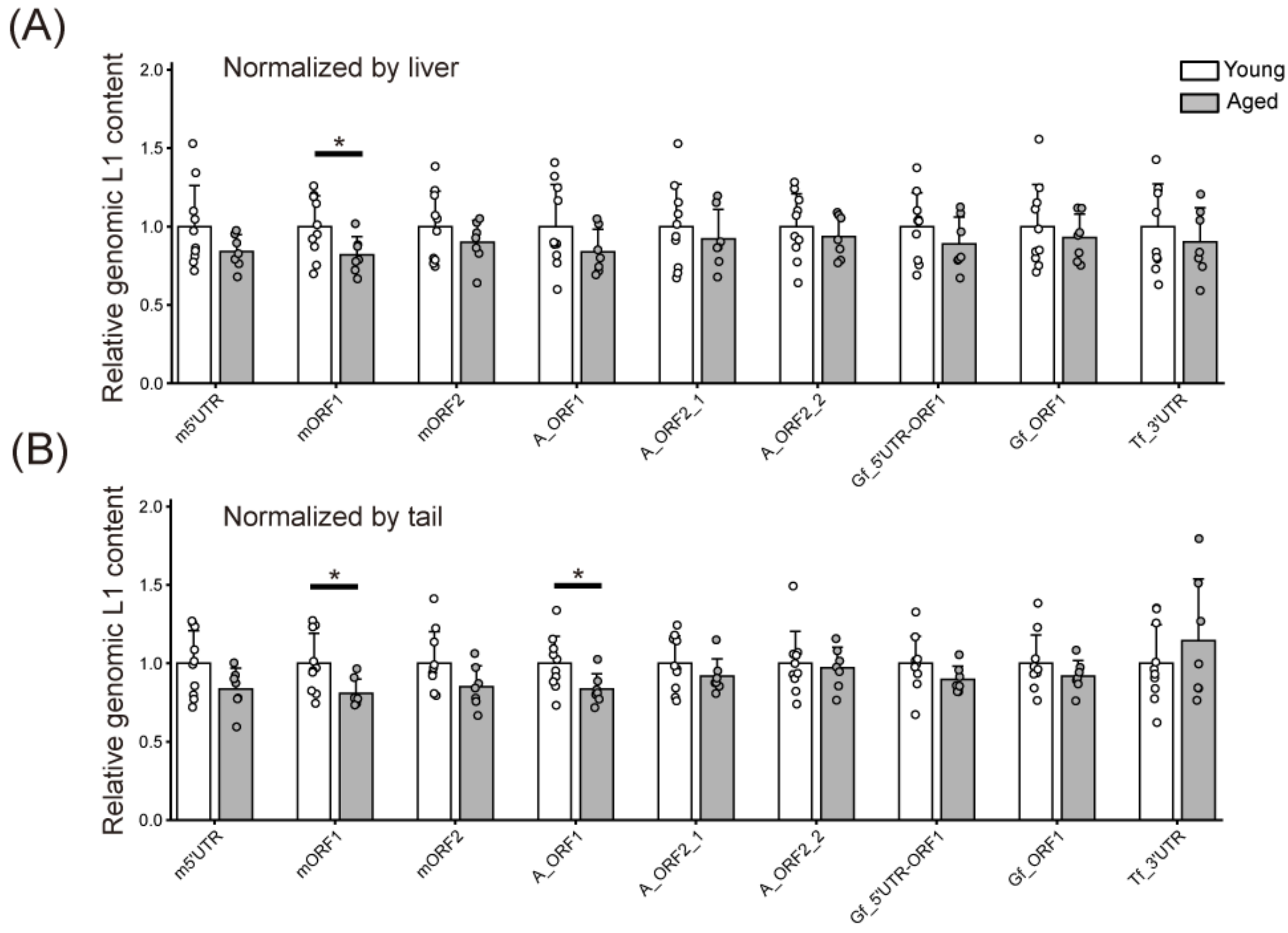

Supplementary Figure 1. Relative L1 genomic copy number in the PFC normalized by liver and tail.

Genomic L1 copy number was quantified by qPCR using primer sets targeting the L1 5′UTR, ORF1, ORF2, and representative active L1 subfamilies (A, Gf, and Tf). The locations of the primer sets used in this study are shown in Figures 1A and 2A. L1 copy number was first normalized to 5S rRNA and subsequently normalized to genomic DNA from the liver (A) or tail (B). Values are expressed relative to the mean of the young group, which was set to 1. Data are presented as mean ± SD with individual animals shown as dots (young, white bars; aged, gray bars). Statistical significance was determined using Welch's t-test. *, $P < 0.05$.

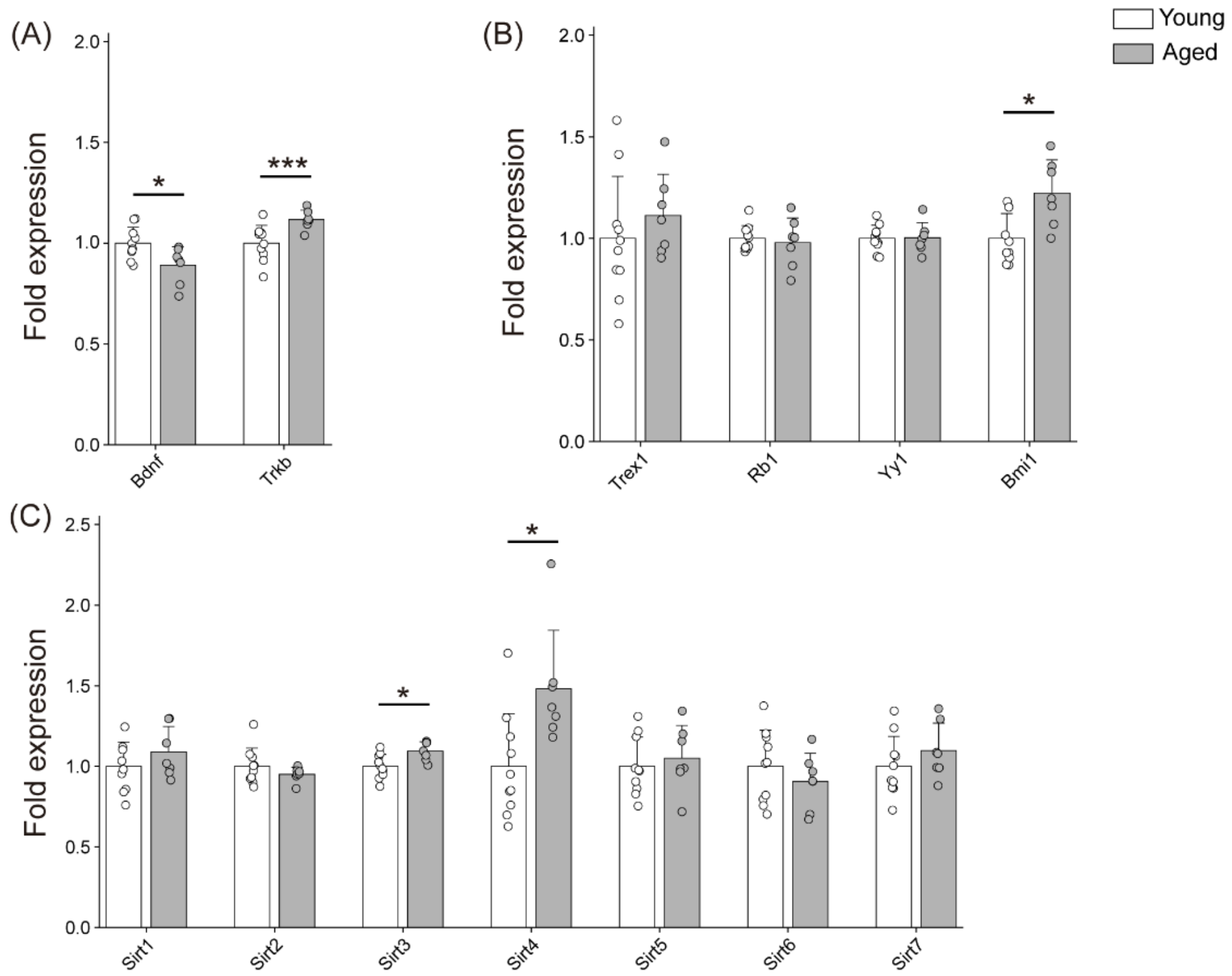

Supplementary Figure 2. RNA-seq confirmation of RT-qPCR findings in the PFC.

RNA-seq analysis of Bdnf and Trkb (A), L1 regulators (B) and Sirt genes (C) in the PFC of young and aged mice. Expression levels were normalized to the mean of the young group, which was set to 1. Data are presented as mean ± SD with individual animals shown as dots (young, white bars; aged, gray bars). Statistical significance was determined using Welch's t-test. *, $P < 0.05$; ***, $P < 0.005$.

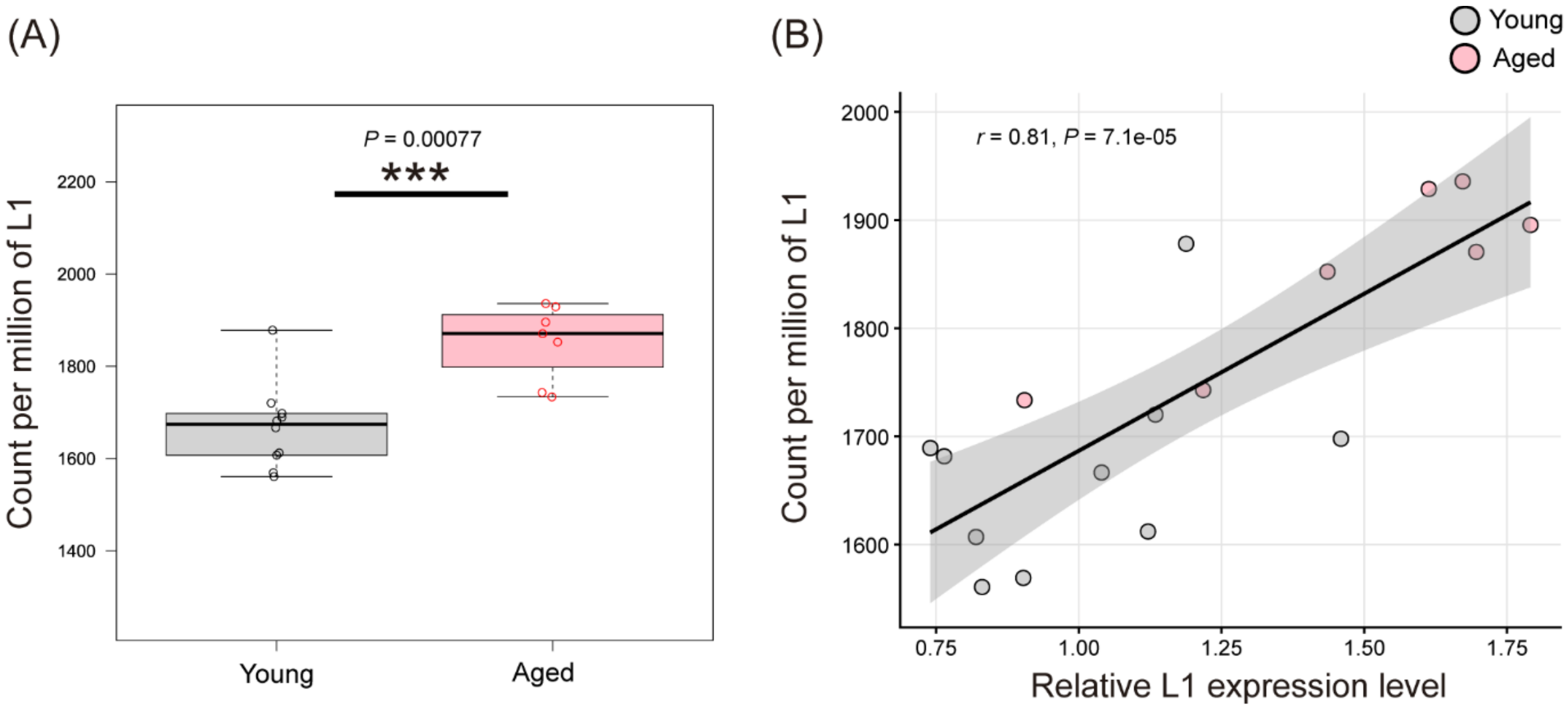

Supplementary Figure 3. RNA-seq confirmation of increased expression of L1.

(A) Whole L1 expression level estimated by TEtranscripts. (B) Correlation of L1 expression level between qPCR and RNA-seq. Individual animals shown as dots (young, white bars; aged, gray bars). Statistical significance in (A) was determined using Student's t-test. ***, $P < 0.005$. Correlation in (B) was determined using Pearson's correlation analysis, $r = 0.81$, $P = 7.1 \times 10^{-5}$.
